# Overexpression of LAPTM4B-35: A Novel Marker of Poor Prognosis of Prostate Cancer

**DOI:** 10.1371/journal.pone.0091069

**Published:** 2014-03-20

**Authors:** Hongtuan Zhang, Qiang Wei, Ranlu Liu, Shiyong Qi, Peihe Liang, Can Qi, Andi Wang, Bin Sheng, Liang Li, Yong Xu

**Affiliations:** 1 Department of Urology, National Key Clinical Specialty of Urology, Second Hospital of Tianjin Medical University, Tianjin Key Institute of Urology, Tianjin Medical University, Tianjin, China; 2 Department of Urology, The Second Affiliated Hospital of Chongqing Medical University, Chongqing, China; 3 Department of Radiology, Second Hospital of Tianjin Medical University, Tianjin Medical University, Tianjin, China; University of Nebraska Medical Center, United States of America

## Abstract

**Background:**

Lysosome-associated protein transmembrane 4b-35 (LAPTM4B-35) is a member of the mammalian 4-tetratransmembrane spanning protein superfamily, which is overexpressed in several solid malignancies. However, the expression of LAPTM4B-35 and its role in the progression of prostate cancer (PCa) is unknown. The aim of the present study was to investigate the LAPTM4B-35 expression in PCa and its potential relevance to clinicopathological variables and prognosis.

**Methods:**

Immunohistochemistry was used to determine the expression of LAPTM4B-35 protein in 180 PCa tissues in comparison with 180 normal benign prostatic hyperplasia (BPH) specimens. The correlation between the expression of the LAPTM4B-35 protein and the clinicopathologic characteristics of patients with PCa was analyzed.

**Results:**

Statistical analysis showed that LAPTM4B-35 expression was significantly elevated in PCa compared with the BPH controls. High LAPTM4B-35 staining was present in 71.11% of all the cases with PCa. The overexpression of LAPTM4B-35 was significantly associated with the lymph node metastasis, seminal vesicle invasion, PCa stage, higher Gleason score, higher preoperative PSA, and biochemical recurrence (BCR). The Kaplan-Meier survival analysis showed that the high expression of LAPTM4B-35 was related to the poor overall survival and BCR-free survival of patients with PCa. Multivariate Cox analysis showed that LAPTM4B-35 was an independent prognostic factor for both overall survival and BCR-free survival of patients with PCa.

**Conclusions:**

Overexpression of LAPTM4B-35 may be associated with tumor progression and poor prognosis in PCa and thus may serve as a new molecular marker to predict the prognosis of PCa patients.

## Introduction

As the most common malignant neoplasm in men, prostate cancer (PCa) is the second most common cause of tumor related deaths in the United States [1]. Although the prognosis for patients with localized or regional disease is good, for patients in the United States who develop metastatic disease, the 5-year survival rate is only 29% [2]. However, the molecular mechanisms underlying carcinogenesis and progression of PCa have not yet been fully explicated. Therefore, efforts to identify more molecular markers that could be used to detect PCa as well as individualize both patient prognosis and therapy are of great clinical importance.

Conventional prognostic factors such as Gleason score, preoperative PSA levels or ratio of involved biopsies only insufficiently predict patient outcome for currently available therapies. They are even more limited in identifying insignificant prostate cancer, i.e. cancer that might be left untreated without shortening the patient's life expectancy but sparing him the morbidity of unwarranted treatment. Therefore, further efforts to find new diagnostic pathways and therapeutic options are urgently demanded to optimise patient management [3]–[6]. The identification of novel prostate cancer biomarkers should be of first clinical priority, when taking into consideration the problematic heterogeneity of prostate tumors and the solutions that personalized medicine can provide. These biomarkers should be capable of enhancing differential diagnosis and indicating the course of PCa disease as early and as accurate as possible, thus ultimately limiting the amount of non-essential medical procedures [7]–[10]. Tissue biomarkers can enrich significantly this approach by providing meaningful information for the management of PCa via the utilization of specimens from biopsies or prostatectomies [8], [11].

Recently, LAPTM4B (lysosomal protein transmem-brane 4A), which was initially identified as a novel gene upregulated in human hepatocellular carcinoma [12], [13], was successfully cloned by fluorescence differential display, rapid amplification of cDNA ends, and reverse transcriptase-polymerase chain reaction. It has been demonstrated that LAPTM4B gene is mapped to chromosome 8q22.1 with an mRNA consisting of 7 exons and encodes a 35-kDa protein, LAPTM4B-35, which is a type-III transmembrane protein with four putative transmembrane regions. Studies have shown that the activity of LAPTM4B-35 is elevated in several human cancers. Moreover, the overexpression of LAPTM4B-35 is associated with a poor prognosis and contributes to cellular transformation, tumorgenesis, and metastatic progression in these cancers [14]–[18]. However, the clinicopathologic significance and biologic role of LAPTM4B-35 in PCa remain unclear.

To date, there have been no published reports evaluating the role of LAPTM4B-35 expression in PCa. Thus, the aim of the present study was to investigate the expression of LAPTM4B-35 in PCa and to analyze its relationship to various clinicopathologic characteristics, including PCa patient outcome.

## Materials and Methods

### Patient and Tissue Samples

Written informed consent was obtained from all of the patients. Approval from the Medical Ethics Committee of the Tianjin Medical University was obtained for the purpose of research (TMUhMEC2012015). Following Institutional Review Board approval, archived formalin-fixed, paraffin-embedded samples were obtained from 180 patients with PCa and 180 patients with benign prostatic hyperplasia (BPH), who were surgically treated in the second hospital of Tianjin Medical University, Tianjin, China between January 1999 and December 2010 [19]–[21]. All tissue specimens and slides were examined by experienced pathologists. None of the patients received chemotherapy, immunotherapy, or radiotherapy before surgery. The clinicopathological features of patients, including preoperative PSA, Gleason score, PCa stage, lymph node status, angiolymphatic invasion status, surgical margin status, seminal vesicle invasion status, and BCR are summarized in Table 1.

**Table 1:** Expression of LAPTM4B-35 in prostate specimens.

		LAPTM4B-35 expression		
Groups	n	High expression	%	P
BPH	180	15	8.33%	<0.001
PCa	180	128	71.11%	

### Immunohistochemical Staining

Four-micrometer sections on the formalin-fixed, paraffin-embedded samples were obtained, followed by hematoxylin and eosin staining for PCa pathology confirmation. We took the sections immediately adjacent the hematoxylin and eosin-stained parts for immunohistochemistry and deparaffinized the selected tissues in xylene and rehydrated with graded alcohol concentrations under standard procedures. Subsequently, the deparaffinized sections were immersed in 0.01 mol/L citrate buffer (pH 6.0) and heated for fifteen minutes in a microwave oven. After sequential incubation with 3% hydrogen peroxide and 10% normal goat serum for ten minutes individually, endogenous peroxidase activity and nonspecific immunoglobulin binding were consecutively blocked in that order. Then, the avidin-biotin immunoperoxidase technique was adopted to perform immunohistochemical staining. Sections were incubated for one hour with primary rabbit anti-human polyclonal antibody LAPTM4B-35 antibody (dilution, 1∶100) (LAPTM4BN199-pAb; Abcam Co) at room temperature, rinsed, and incubated for 30 minutes with biotinylated secondary antibody. After the sections were washed extensively with water, they were incubated with a biotin-labeled secondary antibody followed by horseradish peroxidase-conjugated streptavidin for thirty minutes each. Incubation with 3,3′-diaminobenzidine tetrahydrochloride in 0.01% H2O2 for 10 minutes provided color development. Then, the slides were counterstained with Meyer hematoxylin for thirty to sixty seconds and were mounted in aqueous mounting medium. Finally, immunohistochemistry was performed with an immunohistochemistry kit, following the manufacturer's instructions. Negative control slides were stained with rabbit serum.

### Staining Evaluation

LAPTM4B-35 protein expression levels were classified semiquantitatively based on the total combined scores of the percentage of positive-staining tumor cells and staining intensity [21]. The percentage of positive cells, measured as the extent of immunostaining, was scored as follows: 0, less than 5% staining; 1, 5% to 50%; and 3, >50% staining. The staining intensity was scored as follows: 0, no staining or only weak staining; 1, moderate staining; and 2, strong staining. The staining intensity score plus the percentage of positive staining was used to define the expression levels: 0 to 2, low expression and 3 to 4, high expression [21]. The scoring procedure was carried out in duplicate by 2 independent pathologists who are experienced in evaluating immunohistochemistry and had no knowledge of the clinicopathologic information or the corresponding hematoxylin and eosin slides. The concordance rate between the two primary pathologists was greater than 96%. In cases of significant disagreement, the slides in question were re-reviewed simultaneously by the original two pathologists, together with a third pathologist at a multiheaded microscope, in order to resolve the divergence of opinion.

### Statistical Analysis

The chi-square test was used to analyze the differences of categorical variables. Overall survival and BCR-free survival were calculated according to the Kaplan-Meier method and evaluated by the log-rank test. Cox regression (proportional hazard model) was performed for multivariate analysis of prognostic predictors. P<0.05 was considered significant. Statistical analysis was carried out using SPSS 17.0 software.

## Results

### LAPTM4B-35 protein is overexpressed in PCa tissues

LAPTM4B-35 expression was overexpressed in PCa cases compared with BPH, and the difference was statistically significant (P<0.001) (Table 1). As shown in Figures S1, S2, S3, S4, the LAPTM4B-35 expression was localized in the cytoplasm of prostate cells. Of the 180 specimens with PCa examined, LAPTM4B-35 expression was low in 52 (28.89%) of 180 patients with PCa and high in 128 (71.11%) of 180 patients. LAPTM4B-35 expression was significantly higher in the higher PCa stage and seminal vesicle invasion cases; LAPTM4B-35 expression was also significantly increased in PCa patients with lymph node metastasis, higher preoperative PSA, higher Gleason score, and BCR. Furthermore, no significant association was observed between LAPTM4B-35 immunoreactivity and age, angiolymphatic invasion, and surgical margin status. The association between LAPTM4B-35 expression and clinicopathologic factors is shown in Table 2.

**Table 2:** Clinicopathologic variables and LAPTM4B-35 expression in 180 PCa patients.

		LAPTM4B-35 expression			
Variable	Group	n	High	Low	P value
Age					0.185
	<70	97	73 (75.3%)	24 (24.7%)	
	≥70	83	55 (66.3%)	28 (33.7%)	
Lymph node metastasis					0.028
	Presence	17	16 (94.1%)	1 (5.9%)	
	Absence	163	112 (68.7%)	51 (31.3%)	
Surgical margin status					0.557
	Presence	14	9 (64.3%)	5 (35.7%)	
	Absence	166	119 (71.7%)	47 (28.3%)	
Seminal vesicle invasion					0.034
	Presence	35	30 (85.7%)	5 (14.3%)	
	Absence	145	98 (67.6%)	47 (32.4%)	
Clinical stage					<0.001
	T1	103	62 (60.2%)	41 (39.8%)	
	T2/T3	77	66 (85.7%)	11 (14.3%)	
Preoperative PSA					0.001
	<4	5	1 (20%)	4 (80%)	
	4–10	64	39 (60.9%)	25 (39.1%)	
	>10	111	88 (71.1%)	23 (28.9%)	
Gleason score					0.041
	<7	99	63 (63.6%)	36 (36.4%)	
	7	34	26 (76.5%)	8 (23.5%)	
	>7	47	39 (83.0%)	8 (17.0%)	
Angiolymphatic invasion					0.088
	Presence	35	29 (82.9%)	6(17.1%)	
	Absence	145	99 (68.3%)	46 (31.7%)	
Biochemical recurrence					0.029
	Absence	128	85 (66.4%)	43 (33.6%)	
	Presence	52	43 (82.7%)	9 (17.3%)	

### The impact of LAPTM4B-35 expression on BCR-free survival in PCa

To examine if LAPTM4B-35 expression level is a significant predictor of BCR-free time after radical prostatectomy, Kaplan-Meier curves were plotted between high or low LAPTM4B-35 and BCR-free time. The low LAPTM4B-35 expression had significantly longer BCR-free time after radical prostatectomy compared to patients with high LAPTM4B-35 expression (P<0.001; Figure S5). In univariate analysis, high expression of LAPTM4B-35, higher Gleason score, and seminal vesicle invasion were related to a poor BCR-free survival for patients with PCa. Multivariate analysis showed that high LAPTM4B-35 expression, Gleason score, and seminal vesicle invasion were the independent prognostic factors for BCR-free survival. The results are present in Table 3.

**Table 3:** Prognostic value of LAPTM4B-35 expression for the biochemical recurrence-free survival in univariate and multivariate analyses by Cox regression.

	Univariate analysis			Multivariate analysis		
Covariant	Exp (B)	95% CI	P value	Exp (B)	95% CI	P value
LAPTM4B-35	1.848	1.268–2.692	0.001	2.027	1.386–2.964	<0.001
Gleason score	1.703	1.280–2.265	<0.001	1.828	1.371–2.438	<0.001
Preoperative PSA	1.241	0.705–2.188	0.454			
Age	1.068	0.804–1.419	0.650			
Angiolymphatic invasion	1.084	0.814–1.443	0.580			
Surgical margin status	1.017	0.709–1.459	0.925			
PCa Stage	1.090	0.921–1.291	0.316			
Lymph node metastasis	1.140	0.850–1.528	0.381			
Seminal vesicle invasion	1.505	1.132–2.003	0.005	1.508	1.134–2.007	0.005

### The impact of LAPTM4B-35 expression on overall survival in PCa

To examine the impact of LAPTM4B-35 overexpression on the overall survival, we first performed univariate analysis of traditional clinicopathologic variables for prognosis. Significant variables in the overall survival analysis included LAPTM4B-35 expression, PCa stage, Gleason score, seminal vesicle invasion, and preoperative PSA. Multivariate Cox regression analysis enrolling above-mentioned significant parameters showed that LAPTM4B-35 expression, PCa stage, Gleason score, and preoperative PSA were independent prognostic factors for overall survival of patients with PCa. The results are shown in Table 4.

**Table 4:** Prognostic value of LAPTM4B-35 expression for the overall survival in univariate and multivariate analyses by Cox regression.

	Univariate analysis			Multivariate analysis		
Covariant	Exp (B)	95% CI	P value	Exp (B)	95% CI	P value
LAPTM4B-35	3.108	1.575–6.133	<0.001	2.963	1.483–5.920	<0.001
Gleason score	2.526	1.788–3.568	<0.001	1.915	1.340–2.736	<0.001
Preoperative PSA	2.034	1.338–23.092	0.001	1.997	1.297–3.074	<0.001
Age	1.282	0.917–1.792	0.146			
Angiolymphatic invasion	1.373	0.813–2.319	0.235			
Surgical margin status	1.101	0.703–1.724	0.674			
PCa Stage	4.131	2.888–5.911	<0.001	3.912	2.656–5.761	<0.001
Lymph node metastasis	1.044	0.746–1.462	0.800			
Seminal vesicle invasion	1.358	0.956–1.928	0.087			

## Discussion

In this study, we analyzed LAPTM4B-35 expression by immunohistochemistry in PCa using 180 surgical specimens. We analyzed the association between LAPTM4B-35 expression and traditional clinicopathogical characteristics in PCa. The present data showed that LAPTM4B-35 overexpression was associated with poor survival by analyzing the overall survival and BCR-free survival. High LAPTM4B-35 expression was significantly correlated with higher PCa stage, seminal vesicle invasion, lymph node metastasis, higher preoperative PSA, higher Gleason score, and BCR, but not with age, surgical margin status, and angiolymphatic invasion. Moreover, our data also demonstrated that the patients with high LAPTM4B-35 expression had significantly poor overall survival and BCR-free survival by using the Kaplan-Meier method and log-rank test. Multivariate analysis demonstrated that LAPTM4B-35 expression was an independent prognostic factor for both overall survival and BCR-free survival in patients with PCa. To the best of our knowledge, this is the first study to demonstrate in detail an association of clinicopathologic parameters and prognostic significance of LAPTM4B-35 overexpression in PCa. These results suggest that high LAPTM4B-35 expression plays a critical role in the progression of PCa and is significantly associated with a poor prognosis independently of other factors. This raises the possibility that LAPTM4B-35 may be a prognostic parameter for PCa that is as or more reliable than the clinicopathologic factors currently in use and suggests the possibility to use LAPTM4B-35 in individualization of both patient prognosis and therapy. Our results are consistent with the previous reports of the roles of LAPTM4B-35 in tumor progression, including gallbladder carcinoma, ovarian carcinoma, hepatocellular carcinoma, breast cancer, and extrahepatic cholangiocarcinoma, and support a relationship between high expression of LAPTM4B-35 protein and unfavorable biological behavior in PCa.

However, whether LAPTM4B-35 is effective for clinical application as replacements for, or in addition to, the prognostic parameters currently in use is still unclear, and further investigation is called for to identify whether combined detection of LAPTM4B-35 together with some of these other molecules would be valuable in improving prognostic effectiveness. The close relationship between LAPTM4B-35 overexpression and clinicopathological features predicted that LAPTM4B-35 might play an important role in carcinogenesis and tumor progression. Currently, there have been some clues that are able to help to explain its mechanisms. According to published data from some authors, the overexpression of LAPTM4B-35 results in activation of some protooncogenes, such as c-myc, c-jun, and c-fos, and malignant transformation in some cell lines [22], [23]. So the LAPTM4B gene might serve as a protooncogene through its translation product, LAPTM4B-35. These data provide important evidence to explicate the mechanism by which LAPTM4B expression contributes to carcinogenesis and tumor progression, although the role of LAPTM4B in signal transduction pathways in tumor cells is certainly worth further research.

Furthermore, the significant role of LAPTM4B in cancer suggests the possibility to make it a potential target for anticancer therapies. It has been revealed that transfection of the LAPTM4B gene promoted anchorage-independent growth and colony formation of HLE cells, whereas anti-sense oligonucleotides against LAPTM4B inhibited proliferation of BEL-7402, a hepatocellular carcinoma cell line in which LAPTM4B expression was found. Moreover, a high homology to LAPTM4A indicated that LAPTM4B might have a similar function in multidrug resistance [24], [25]. Moreover, the important role of LAPTM4B in cancer suggests the possibility to make it a potential target for anticancer therapies. Some previous studies indicated that LAPTM4B may be a new target of therapy. All these results indicate the possibility to make LAPTM4B as a potential target for therapy of PCa. Definitely, further strong supports from basic investigations are needed.

Our study has some limitations. The sample size is not large enough. To solve this problem, a randomized study investigating the association between LAPTM4B-35 expression and prognosis should be conducted to confirm whether LAPTM4B-35 could be used as a novel predictor of PCa prognosis. In summary, this is the first study to show an association between LAPTM4B-35 overexpression and PCa. We demonstrated that LAPTM4B-35 is overexpressed in a great proportion of patients with PCa, and high LAPTM4B-35 expression correlated with PCa progression and poor prognosis in PCa. Furthermore, LAPTM4B-35 might be an attractive target for PCa therapy. However, these findings remain to be confirmed by future studies.

## Supporting Information

Figure S1 **Representative photograph showing high LAPTM4B-35 expression in PCa by immunohistochemistry.** Original magnification, ×200.
(TIF)

Figure S2 **Representative photograph showing low LAPTM4B-35 expression in PCa by immunohistochemistry.** Original magnification, ×200.
(TIF)

Figure S3 **Representative photograph showing low LAPTM4B-35 expression in BPH by immunohistochemistry.** Original magnification, ×200.
(TIF)

Figure S4 **Representative photograph showing no LAPTM4B-35 expression in PCa by immunohistochemistry (negative control).** Original magnification, ×200.
(TIF)

Figure S5 **Associations between LAPTM4B-35 expression and BCR-free time after radical prostatectomy in PCa patients.** Patients with high LAPTM4B-35 expression showed significantly shorter BCR-free survival than those with low LAPTM4B-35 expression (P<0.001, log-rank test).
(TIF)
