# Supplementary figures and images for: Overexpression of LAPTM4B-35: A Novel Marker of Poor Prognosis of Prostate Cancer
Source: PLoS One. 2014 Mar 20;9(3):e91069. doi: 10.1371/journal.pone.0091069 (PMC3961215; doi:10.1371/journal.pone.0091069)

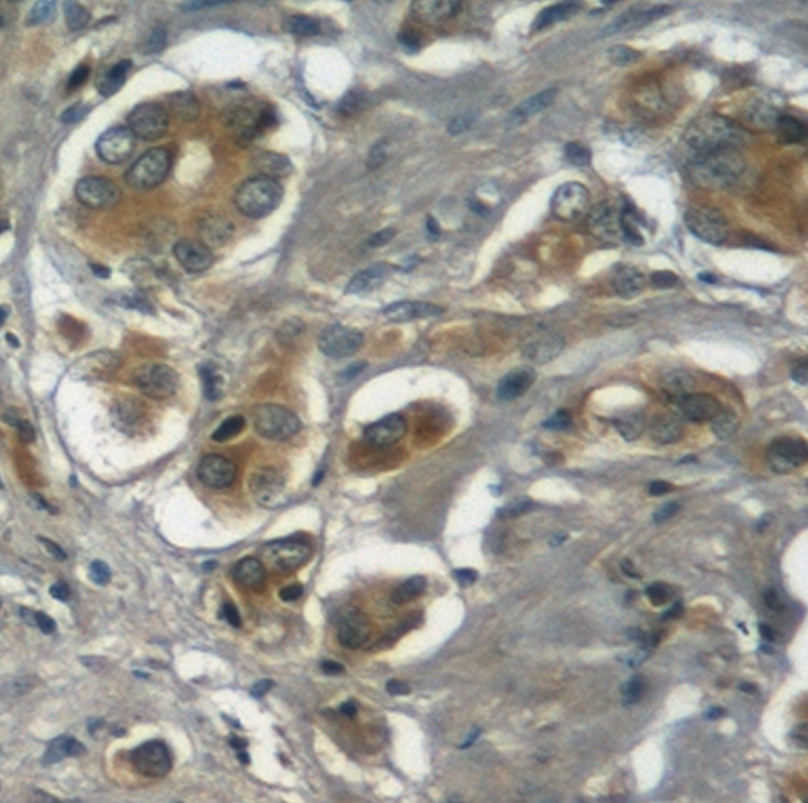

Supplement: Figure S1 — Representative photograph showing high LAPTM4B-35 expression in PCa by immunohistochemistry. Original magnification, ×200. (TIF) [file pone.0091069.s001.tif]

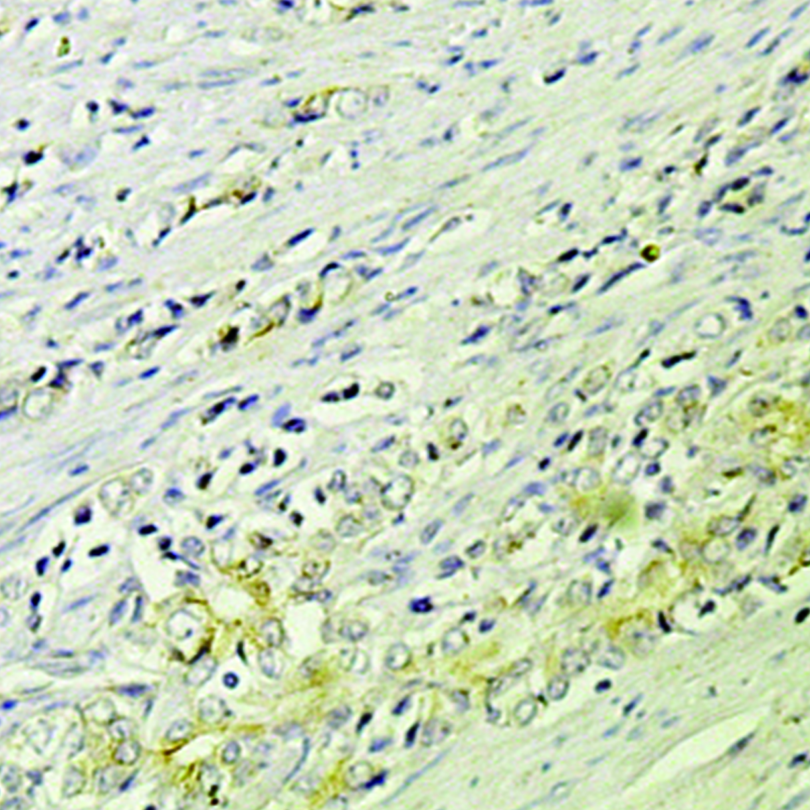

Supplement: Figure S2 — Representative photograph showing low LAPTM4B-35 expression in PCa by immunohistochemistry. Original magnification, ×200. (TIF) [file pone.0091069.s002.tif]

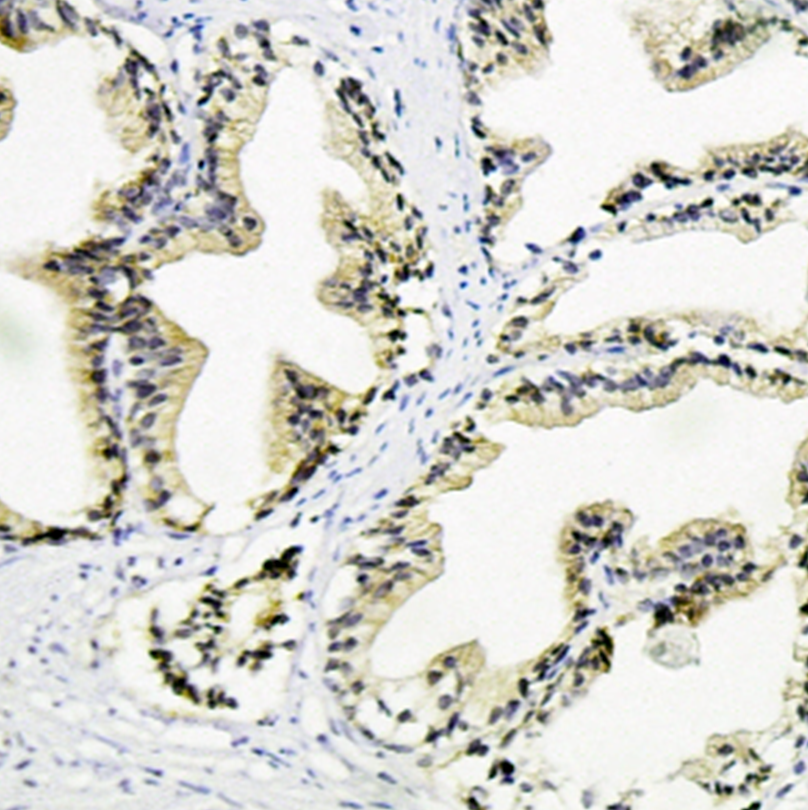

Supplement: Figure S3 — Representative photograph showing low LAPTM4B-35 expression in BPH by immunohistochemistry. Original magnification, ×200. (TIF) [file pone.0091069.s003.tif]

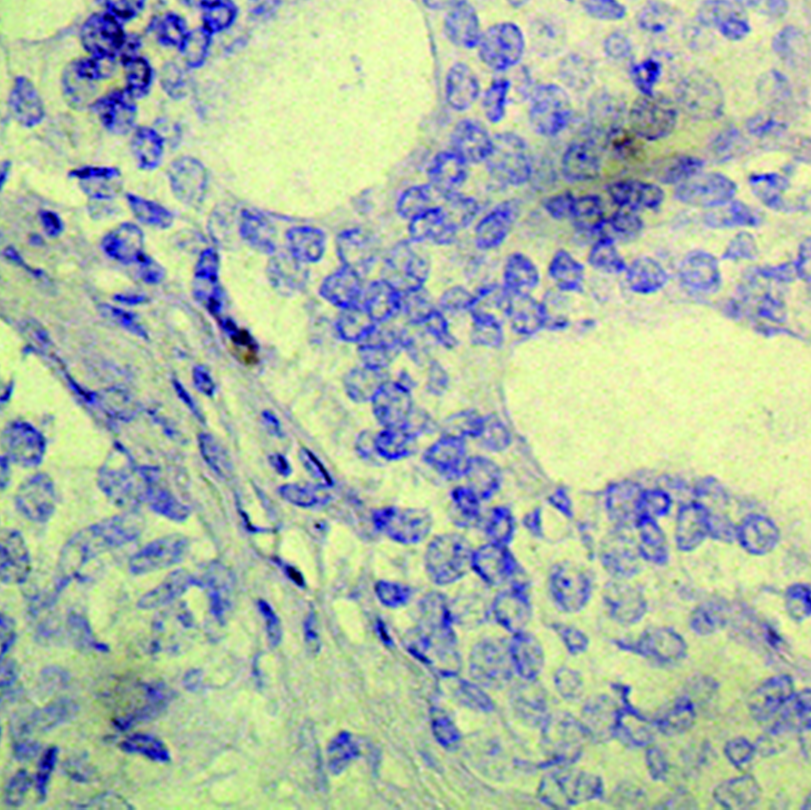

Supplement: Figure S4 — Representative photograph showing no LAPTM4B-35 expression in PCa by immunohistochemistry (negative control). Original magnification, ×200. (TIF) [file pone.0091069.s004.tif]

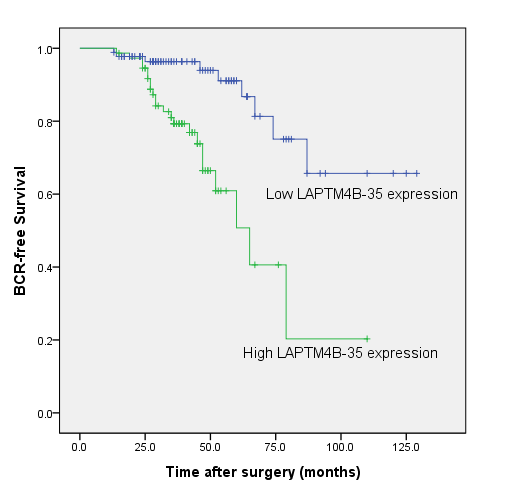

Supplement: Figure S5 — Associations between LAPTM4B-35 expression and BCR-free time after radical prostatectomy in PCa patients. Patients with high LAPTM4B-35 expression showed significantly shorter BCR-free survival than those with low LAPTM4B-35 expression (P<0.001, log-rank test). (TIF) [file pone.0091069.s005.tif]
